# Quantification of left ventricular contribution to stroke work by longitudinal and radial force-length loops

**DOI:** 10.1152/japplphysiol.00198.2020

**Published:** 2020-08-20

**Authors:** Felicia Seemann, Jonathan Berg, Kristian Solem, Robert Jablonowski, Håkan Arheden, Marcus Carlsson, Einar Heiberg

**Affiliations:** ^1^Department of Clinical Sciences Lund, Clinical Physiology, Lund University, Skåne University Hospital, Lund, Sweden; ^2^Department of Biomedical Engineering, Faculty of Engineering, Lund University, Lund, Sweden; ^3^Syntach AB, Lund, Sweden; ^4^Wallenberg Center for Molecular Medicine, Lund University, Lund, Sweden

**Keywords:** force-length loops, longitudinal pumping, radial pumping, stroke work, ventricular forces

## Abstract

Left ventricular (LV) stroke work (SW) is calculated from the pressure-volume (PV) loop. PV loops do not contain information on longitudinal and radial pumping, leaving their contributions to SW unknown. A conceptual framework is proposed to derive the longitudinal and radial contributions to SW, using ventricular force-length loops reflecting longitudinal and radial pumping. The aim of this study was to develop and validate this framework experimentally and to explore these contributions in healthy controls and heart failure patients. Thirteen swine underwent cardiovascular magnetic resonance (CMR) and LV pressure catheterization at baseline (*n* = 7) or 1 wk after myocardial infarction (*n* = 6). CMR and noninvasive PV loop quantification were performed on 26 human controls and 14 patients. Longitudinal and radial forces were calculated as LV pressure multiplied by the myocardial surface areas in the respective directions. Length components were defined as the atrioventricular plane and epicardial displacements, respectively. Contributions to SW were calculated as the area within the respective force-length loop. Summation of longitudinal and radial SW had excellent agreement with PV loop-derived SW (ICC = 0.95, *R* = 0.96, bias ± SD = −4.5 ± 5.4%) in swine. Longitudinal and radial contributions to SW were ~50/50% in swine and human controls, and 44/56% in patients. Longitudinal pumping required less work than radial to deliver stroke volume in swine (6.8 ± 0.8 vs. 8.7 ± 1.2 mJ/mL, *P* = 0.0002) and in humans (11 ± 2.1 vs. 17 ± 4.7 mJ/mL, *P* < 0.0001). In conclusion, longitudinal and radial pumping contribute ~50/50% to SW in swine and human controls and 44/56% in heart failure patients. Longitudinal pumping is more energy efficient than radial pumping in delivering stroke volume.

**NEW & NOTEWORTHY** A novel method for quantifying the contributions of longitudinal and radial pumping to stroke work using global left ventricular force-length loops was proposed and validated, which can be quantified noninvasively using cardiovascular magnetic resonance and brachial cuff pressure. We found that longitudinal and radial pumping contributes equally to stroke work in controls and 44/56% in heart failure patients, and that the longitudinal pumping is more energy efficient in delivering stroke volume than radial pumping.

## INTRODUCTION

Ventricles are a syncytium of branched myocytes with a complex obliquely oriented and helical microstructure that reorients and deforms during contraction (18, 27). Ventricular pumping is the result of contractile forces developed locally in the myocytes, together contributing to global ventricular shortening (22). The global deformation and displacement of the myocardial tissue can from a simplified perspective be observed and fractioned into two main comprehensive global modes of pumping: longitudinal and radial pumping (15, 22, 46). Longitudinal pumping refers to the displacement of the atrioventricular (AV) valve plane in the apical-basal direction, while radial pumping refers to the inward epicardial displacement (5, 42). Importantly, the division into longitudinal and radial components is the result of myocardial shortening in three dimensions and includes helical twisting and sheetlet reorientation. From an outward perspective, the volume changes can be seen as longitudinal and radial displacements that are the result of the more complex myocardial rearrangement. The division into systolic volume displacements has physiological significance for the systolic atrial filling, which is the result of longitudinal (AV-plane) ventricular pumping. The portion of left ventricular (LV) stroke volume accounted for by longitudinal pumping is ~60%, while the portion accounted for by radial pumping is ~40% (5, 6). Previous work has also shown that the LV AV-plane displacement, also known as mitral annular plane systolic excursion (MAPSE), is a parameter of ventricular function that provides complementary information to ejection fraction (16, 17) and is furthermore an independent predictor of major cardiac events and mortality (3, 30, 31, 33, 47).

Although stroke volume is an important variable for the evaluation of ventricular function, it does not contain information on cardiac energy consumption. The portion of the ventricular energy consumption required to eject blood corresponds to the stroke work (SW), and is calculated as the area within the ventricular pressure-volume loop (4). However, the pressure-volume loop does not inherently contain information on longitudinal and radial pumping, as pressure and volume are scalar quantities without directional components. Hence, the quantitative longitudinal and radial contributions to SW are unknown.

Work with directional components can instead be quantified from force-length loops, as forces are vectors with inherent magnitude and direction. Although the force-length loop of isolated cardiac muscle strips has been extensively studied (7, 19, 23), the corresponding translation into global ventricular loops is lacking, because force cannot be directly measured in the intact ventricle. Pressure-strain loop analysis has instead been proposed as an approximated index of regional work (8, 32, 41, 43). Global ventricular force-length loop assessments have, however, been studied only in model-based simulations (29). In this study, we propose a simplified conceptual framework to derive two subject-specific global ventricular force-length loops. The force-length loops are derived using LV geometrical assessments by cardiovascular magnetic resonance (CMR), thus fractioning the pressure-volume loop-derived SW into one longitudinal and one radial component. The forces exerted on the ventricular wall due to blood pressure are calculated using the fundamental physical relationship among force, pressure, and area. Length is derived from the global myocardial tissue displacement in the longitudinal and radial directions, thus creating two force-length loops.

We hypothesized that the sum of the areas within these longitudinal and radial force-length loops equals the stroke work calculated from pressure-volume loops, meaning that the longitudinal and radial contributions to LV SW can be calculated from force-length loops. Therefore, the aims of this study were *1*) to develop and experimentally validate a conceptual framework for ventricular force-length loops in a swine model and *2*) to explore the longitudinal and radial contributions to LV SW in healthy human controls and patients with heart failure due to dilated ischemic cardiomyopathy.

## MATERIALS AND METHODS

### 

#### Experimental protocol.

The experimental protocol was submitted to and approved by the Swedish Agricultural Board. Thirteen swine were used [10 male, 47 ± 7 kg, *Sus scrofa domesticus*), of which nine have had results from experiments previously published (36)]. Seven experiments were performed at baseline and six experiments 1 wk postinduction of myocardial infarction by a 40-min balloon occlusion in the left anterior descending artery (28). Baseline experiments and the induced myocardial infarctions were not performed on the same animals, meaning that swine were not their own controls.

The study protocol included a CMR examination and invasive LV blood pressure measurements, which were performed within 3 h of each other. Anesthesia was maintained on isoflurane gas during the entire protocol.

CMR cine images in the long-axis 2-chamber, 3-chamber, and 4-chamber views as well as a short-axis stack covering the entire left ventricle were acquired with a balanced steady-state-free-precession (bSSFP) sequence on a 1.5 T Siemens area system (Siemens, Erlangen, Germany) at end expiration with retrospective echocardiogram (ECG) gating. Typical spatial resolution was 1.5 × 1.5 × 8 mm with no slice gap, and temporal resolution was 31 ms with 25 reconstructed time frames, echo time 1.1 ms, flip angle 60°, and field of view 270 × 320 mm^2^.

Blood pressure measurements consisted of 3- to 17-min recordings of invasive LV pressure using a 5F pressure-volume conductance catheter (Transonic, Ithaca, NY) inserted through the aortic valve via the femoral artery.

#### Human cohort.

Inclusion protocols were submitted to and approved by the regional Ethics Review Board in Lund, Sweden, and all study participants provided written, informed consent. Patients from the CRT-CLINIC study (NCT01426321) were considered for this study. The CRT-CLINIC study included advanced heart failure patients with an ejection fraction (EF) <35% confirmed by echocardiography, who were scheduled for cardiac resynchronization therapy. Fourteen patients (69 ± 8 yr, 1 female) who met the criterion of dilated ischemic cardiomyopathy with a successful preoperative CMR at either 1.5 or 3 T (Philips Healthcare, Best, The Netherlands) were included. Furthermore, 26 healthy controls imaged at either 1.5 or 3 T (Philips Healthcare or Siemens) were retrospectively included. The healthy controls were divided into two cohorts, one cohort of 12 young participants (29 ± 8 yr, 4 females), and one older cohort of 14 participants that were age-matched to the patients (69 ± 9 yr, 7 females).

Cine images of the long-axis 2-chamber, 3-chamber, and 4-chamber and a short-axis stack covering the LV were acquired at end expiration with bSSFP and retrospective ECG gating. Typical spatial resolution was 1.5 × 1.5 × 8 mm with no slice gap, and temporal resolution was 30 ms with 30 reconstructed time frames, echo time 1.4 ms, flip angle 60°, and field of view 350 × 350 mm^2^. Noninvasive systolic and diastolic brachial pressure was measured in conjunction with the CMR examination, using cuff sphygmomanometry and a stethoscope for Korotkoff sound detection.

#### Image analysis.

Image analysis was performed semiautomatically using the freely available software Segment (http://segment.heiberg.se) (14), with manual corrections as needed. LV epicardial and endocardial borders were delineated in all slices and time frames in the short-axis images and were used to calculate LV volume, surface area, and radial displacement continuously over the cardiac cycle. Stroke volume was calculated as the difference between end-diastolic and end-systolic volumes. All volume, area, and displacement measurements were interpolated to 100 time points, and smoothing was performed using a three-point sliding mean to avoid discontinuities. The consistency of the LV contours was verified by computing the LV mass in each timeframe, as myocardial mass is conserved over the cardiac cycle.

**Fig. 1.**
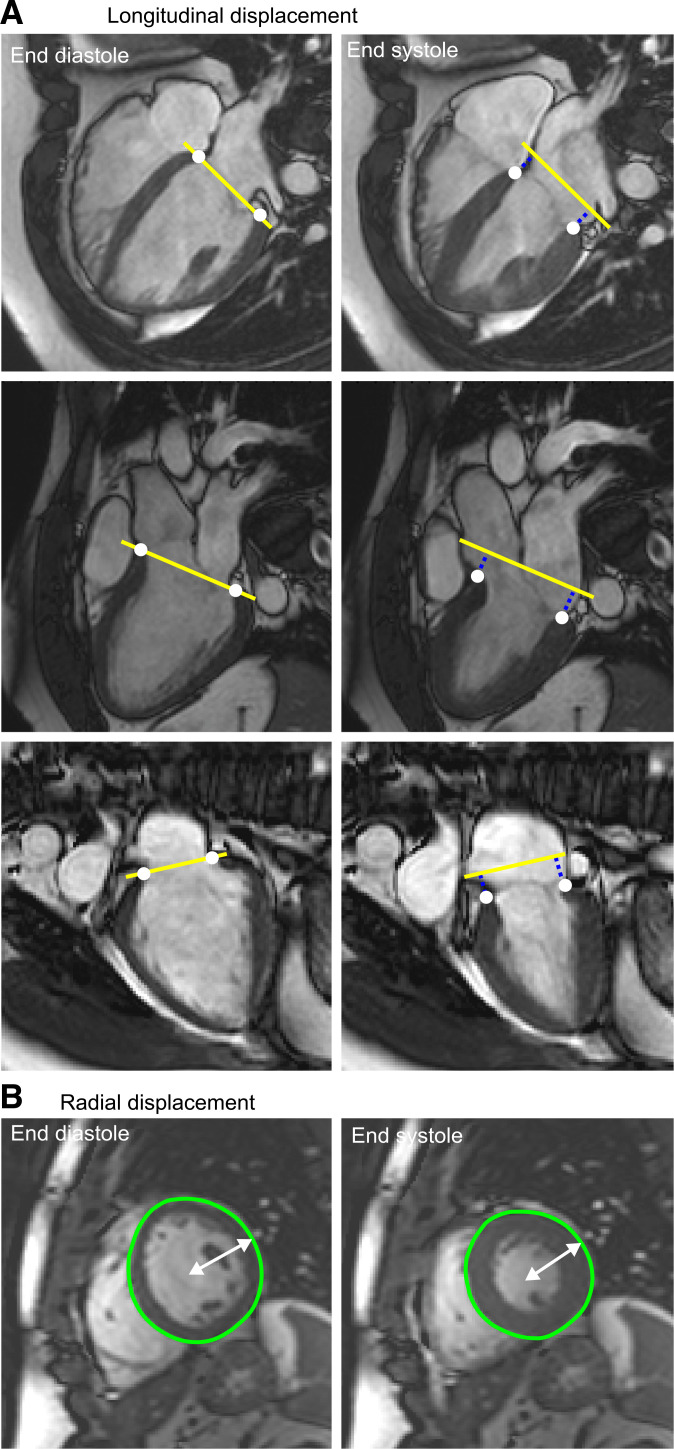
Longitudinal and radial length calculations. *A*: longitudinal displacement was defined as the atrioventricular (AV) plane displacement measured from long-axis images. Basal left ventricular (LV) myocardial insertion points (white dots) were tracked in all time frames. Perpendicular distance (blue dotted lines) from the end-diastolic atrioventricular plane (yellow line) to the points was calculated. The average displacement of the 6 points was considered the global longitudinal displacement. *B*: radial displacement was calculated as change in radius (white) of epicardial delineations (green) in each short-axis slice.

The overall process for calculating the displacements is illustrated in Fig. 1. Longitudinal displacement was calculated as the AV-plane displacement, which was measured in the long-axis images by performing feature tracking of six basal LV myocardial insertion points in all time frames and subsequently averaging the perpendicular displacement of all points relative to the AV plane at end diastole (37). Radial displacement was calculated as the difference between epicardial radius and end-diastolic radius. The epicardial delineations in each slice consisted of 80 points, with a center point defined as the mean of their *x-* and *y*-coordinates. Radii were calculated as the average distance between the center point and the delineated epicardial points.

#### Left ventricular pressure.

In the experimental setup, the average pressure curve over all heartbeats was calculated from the invasive-pressure recordings, interpolated to 100 points, and synchronized in time to the LV volume measured from CMR. In human controls and patients, a recently-proposed and validated method for noninvasive calculation of LV pressure was used (36). In short, a time-varying elastance model is subject-specifically scaled using time-resolved LV volume delineations from CMR and brachial cuff pressure, together with a user estimation of end-diastolic LV pressure. End-diastolic LV pressure was unknown in the human cohort in this study and was set to 5 mmHg, and the equilibrium volume V_0_ was approximated to zero.

#### Longitudinal and radial forces.

The contracting forces in the myocardial wall generate blood pressure. As Newton’s third law implies, if the myocardium exerts a force on the blood, then the blood exerts a counteracting balancing force on the myocardium. The balancing force F generated by the blood pressure P onto the myocardium was calculated as

**Fig. 2.**
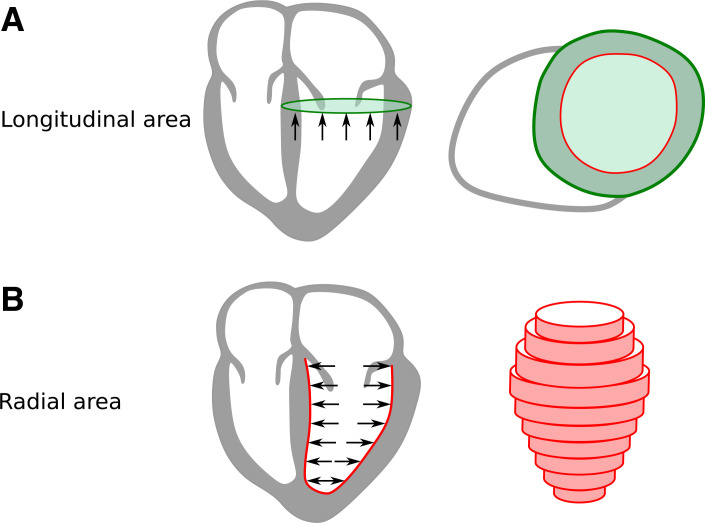
Schema of areas on which longitudinal and radial forces act. *A*: longitudinal forces act on the largest cross-sectional epicardial area (green). *B*: radial forces, illustrated by black arrows, act upon endocardial surface area, calculated in each short-axis slice as circumference multiplied by slice thickness (red).

**Fig. 3.**
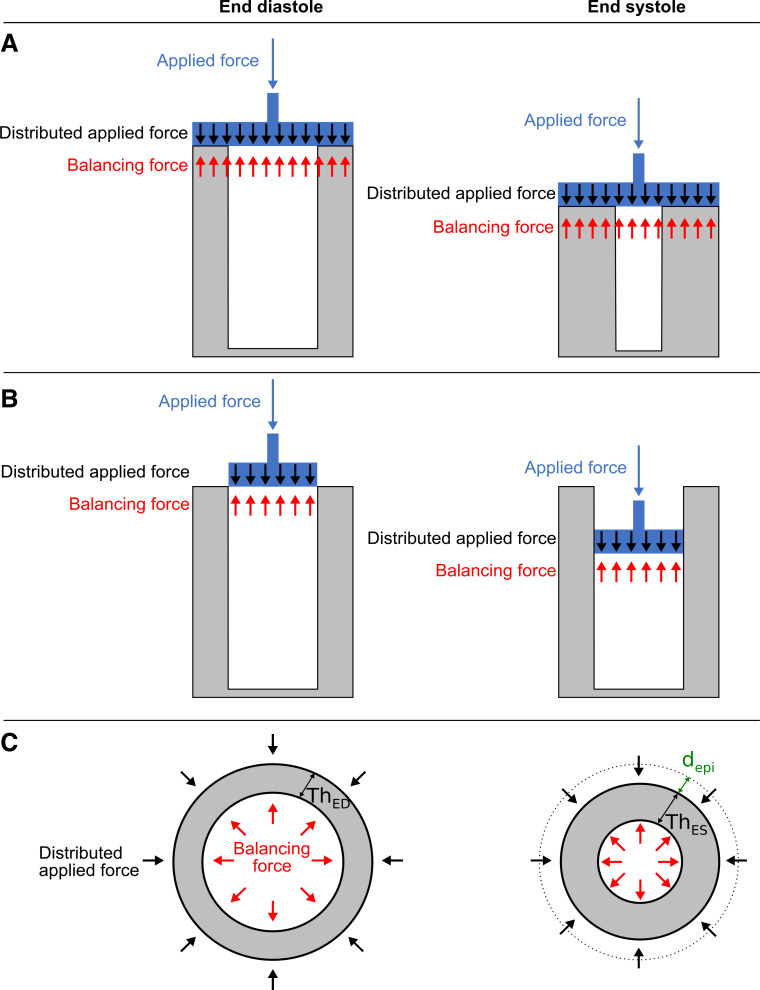
Schema of simplified cylindrical ventricular model describing the rationale behind the definition of the epicardial cross-sectional area as the surface area on which the longitudinal forces act and the epicardial displacement as the radial length component. *A*: illustration of an isolated longitudinal contraction using the epicardial cross-sectional area. The myocardial wall is highlighted in gray, and an imaginary piston applying an external force on the myocardium is shown in blue. The applied force on the piston handle (blue arrow) will distribute itself over the piston area (black arrows). These forces will generate a pressure inside the ventricle, which will then experience balancing forces along the contact area of the piston according to Newton’s third law. Due to conservation of myocardial mass, the longitudinal contraction results in wall thickening between end diastole and end systole. *B*: illustration of how a model only accounting for the endocardial surface area would impact the myocardium. As both the myocardium and the blood pool experience shortening in this direction, it follows that the piston covers the entire epicardial cross-sectional area as illustrated in *A*. Longitudinal forces acting on the endocardial cross-sectional area alone, as in *B*, would result in the piston sliding along the endocardium, shortening the blood pool but not the myocardium, which would remain in the exact same shape and position, without any wall thickening, over the cardiac cycle. *C*: illustration of an isolated radial contraction in the short-axis perspective, with a fixed cylinder height. Black arrows illustrate distributed applied forces in the radial direction along the epicardial border, which generate blood pressure inside the ventricle, thus inducing balancing radial forces along the endocardium. The distributed applied forces also generate tissue displacement. At end systole, the outer epicardial boundary will have experienced a displacement of length, d_epi_, illustrated in green, as the distance between the end-diastolic (dotted line) and end-systolic epicardial borer. Due to mass conservation of the myocardial tissue, it follows that the end-systolic myocardial wall thickness, Th_ES_, is larger than the end-diastolic myocardial wall thickness, Th_ED_. The endocardial displacement does therefore reflect both tissue displacement and wall thickening, whereas the epicardial displacement reflects only the tissue displacement. Taking longitudinal and radial contractions together, it follows that the endocardial displacement reflects a combination of wall thickening from both modes of pumping as well as the radial displacement.

F=P×A

where *A* is the surface area on which the force acts (13). Radial force was defined as the LV pressure multiplied by the endocardial surface area in each time frame. The endocardial surface area was calculated in each short-axis slice as the circumference of the endocardial delineation multiplied by the slice thickness, as shown in Fig. 2. The longitudinal force was defined as the LV pressure multiplied by the maximum epicardial cross-sectional area, which was calculated from the short-axis slice with the largest cross-sectional area at each point in time. The reason for considering the epicardial area rather than the endocardial area for longitudinal pumping is illustrated in a geometrical model in Fig. 3, *A* and *B*, and was based on how the myocardium and blood pool shortens in the longitudinal direction.

#### Force-length loops.

The longitudinal force-length loop was defined by the longitudinal force and the AV-plane displacement, and the area within this loop corresponds to the longitudinal contribution to SW.

The radial force-length loop was defined by the radial force and the epicardial displacement. The rationale for considering the epicardial rather than endocardial displacement is illustrated in Fig. 3*C*, which is more suitable than the endocardial displacement, which, besides radial displacement, also reflects wall thickening due to both the longitudinal and radial modes of pumping. Due to the elliptical shape of the left ventricle, radial force-length loops were calculated separately for each depicted short-axis slice. Thus, one force-length loop was calculated for each slice over all time frames, and the total radial contribution to SW was calculated as the sum of the areas within each slice-loop.

#### Stroke work.

Total ventricular SW was calculated as the sum of the longitudinal and radial contributions to SW, derived from the separately calculated areas within each respective force-length loop. This calculation was validated by conventional computation of SW as the area within the pressure-volume loop. Pressure-volume loops were derived using LV volume from CMR images and LV pressure. The longitudinal and radial contributions to SW as a percentage of total SW were calculated.

#### Work per ejected volume.

Longitudinal contribution to stroke volume (SV_long_) was calculated as the maximum end-systolic AV-plane displacement multiplied by the mean of the two largest basal epicardial short-axis areas at end diastole, as previously described (6). Radial contribution to stroke volume (SV_rad_) was calculated as the remaining amount of total stroke volume. The performed work per ejected volume (WEV, in mJ/mL) defined as the fraction of SW and stroke volume was calculated for longitudinal and radial pumping as

$${\text{WEV}}_{\text{long}}=\frac{{\text{SW}}_{\text{long}}}{{\text{SV}}_{\text{long}}}$$

and

$${\text{WEV}}_{\text{rad}}=\frac{{\text{SW}}_{\text{rad}}}{{\text{SV}}_{\text{rad}}}$$

respectively. WEV_long_ and WEV_rad_ can be considered the work required to eject 1 mL of blood from the left ventricle to the aorta, thus representing the energy efficiency in delivering stroke volume for each respective mode of pumping.

#### Sensitivity analysis.

The equilibrium volume V_0_ was approximated to zero in the noninvasive pressure-volume loop calculations. Previous work has, however, found that V_0_ increases for each *New York Heart Association* functional class in patients with chronic heart failure (20), to values well above zero. A sensitivity analysis was therefore performed to assess how much the parameter V_0_ impacts the noninvasive quantitative stroke work estimation, as well as the relative longitudinal and radial contributions to stroke work in percent. Total, longitudinal, and radial SW was calculated for all integer values of V_0_ ranging between 0 and 90% of the end systolic volume (ESV) in the heart failure patients.

#### Implementation.

A plug-in user interface for force-length loop calculation was implemented in the software Segment and may be made available upon reasonable request.

#### Statistical analysis.

Statistical analysis was performed in GraphPad 8.4.2 (GraphPad. Inc., La Jolla, CA). Mann–Whitney tests were used to compare parameters between the swine cohorts, and Kruskal–Wallis tests were used to compare parameters between young healthy controls, age-matched healthy controls, and patients. Comparisons of longitudinal and radial contributions to SW and WEV within the same cohort were performed with *t* tests. Agreement was assessed by Bland–Altman analysis and intraclass correlation coefficient (ICC). Levels of agreement for ICC were defined as poor (0.00–0.30), weak (0.31–0.50), moderate (0.51–0.70), strong (0.71–0.90), and excellent (0.91–1.00) (21). Pearson *R* were reported, and statistical significance was considered as *P* < 0.05.

## RESULTS

**Fig. 4.**
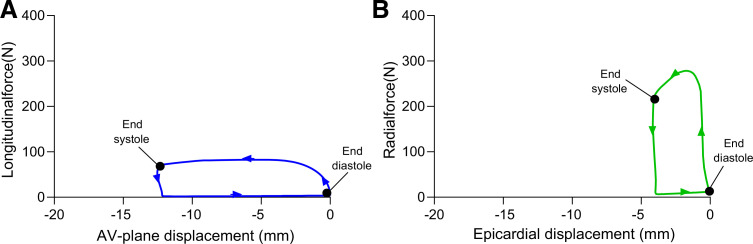
Example of corresponding longitudinal (blue; *A*) and radial (green; *B*) force-length loops in a heart failure patient. Displacements are displayed as negative values so that the loops can be interpreted in the same way as a pressure-volume loop. End diastole occurs in the *bottom right* corner at displacement zero. Moving counterclockwise, as indicated by the arrows, end diastole is followed by a steep increase in force during the isovolumetric contraction, which decreases during ejection. The *top left* corner denotes end systole, which is followed by a fast decrease in force during the isovolumetric relaxation. In diastole, the tissue returns to its original position at displacement zero. The considerably higher peak radial force compared with the peak longitudinal force proceeds from the larger radial surface area, as both surfaces experience the same pressure. AV, atrioventricular.

Characteristics of the human participants and swine are presented in Tables 1 and 2. LV mass variation over the cardiac cycle was 0.06 ± 1.82 and 0.36 ± 2.28% in the experimental and human cohorts, respectively. The low variation indicates consistent delineations, as the myocardial mass is conserved over the heartbeat. Examples of generated longitudinal and radial force-length loops in a patient are shown in Fig. 4. The force-length loops are to be interpreted as a pressure-volume loop moving counterclockwise over the heartbeat, with end diastole in the *bottom right* corner and end systole in the *top left* corner.

**Table 1.** Human participant characteristics

	Young Healthy Controls (*n* = 12)	Age-Matched Healthy Controls (*n* = 14)	Patients (*n* = 14)
Age, yr	29 ± 8	69 ± 9*	69 ± 8*
Body surface area, m^2^	1.9 ± 0.2	1.8 ± 0.2	2.1 ± 0.2†
Heart rate, beats/min	60 ± 8.2	60 ± 8.5	64 ± 9.9
Systolic blood pressure, mmHg	119 ± 15	130 ± 10	125 ± 25
Diastolic blood pressure, mmHg	74 ± 11	75 ± 9	75 ± 14
LV end-diastolic mass, g	115 ± 22	125 ± 34	170 ± 39*†
LV end-systolic mass, g	115 ± 23	123 ± 32	170 ± 38*†
LV end-diastolic volume, mL	196 ± 46	151 ± 30	295 ± 72*†
LV end-systolic volume, mL	81 ± 25	61 ± 14	202 ± 64*†
LV stroke volume, mL	115 ± 25	90 ± 18*	93 ± 20
Longitudinal contribution to SV, mL	66 ± 14	56 ± 11	50 ± 12*
Longitudinal contribution to SV, %	58 ± 6.9	63 ± 6.4	55 ± 7.6†
Radial contribution to SV, mL	49 ± 16	34 ± 10*	42 ± 11
Radial contribution to SV, %	42 ± 6.9	37 ± 6.4	45 ± 7.6†
Ejection fraction, %	59 ± 4.6	59 ± 4.0	32 ± 7.8*†
Cardiac output, L/min	6.9 ± 1.8	5.3 ± 1.1*	5.9 ± 1.4
Rate pressure product, mmHg/min	7,204 ± 1,513	7,873 ± 1,515	8,172 ± 2,436
Max AV-plane displacement, mm	16 ± 2.2	14 ± 1.7	8.3 ± 2.0*†
Max average epicardial displacement, mm	2.4 ± 0.4	2.4 ± 0.5	1.7 ± 0.5*†
End-diastolic longitudinal epicardial surface area, cm^2^	44 ± 6.4	41 ± 5.7	63 ± 9.1*†
End-diastolic radial endocardial surface area, cm^2^	153 ± 24	128 ± 18	190 ± 29*†
Peak LV pressure (noninvasive calculation), mmHg	107 ± 18	119 ± 9.1	116 ± 22
Cause of IHD, *n* (%)	0 (0)	0 (0)	14 (100)
LBBB, *n* (%)			14 (100)
Diabetes, *n* (%)			2 (14)
NYHA class			3 ± 0.8
Medication, *n* (%)	0 (0)	0 (0)	14 (100)
β-blockers, *n* (%)			13 (93)
ACE inhibitors or ARBs, *n* (%)			14 (100)
Thrombocyte aggregation inhibitors, *n* (%)			10 (71)
Diuretics, *n* (%)			13 (93)
Statins, *n* (%)			12 (86)

Continuous variables are reported as means ± SD. Ischemic heart disease (IHD) was determined by a significant amount of myocardial scar measured with late gadolinium-enhanced cardiovascular magnetic resonance imaging. LV, left ventricular; LBBB, left bundle branch block; NYHA, New York Heart Association classification; ACE, angiotensin-converting enzyme; ARB, angiotensin II receptor blocker.

* Significant differences (*P* < 0.05) from young healthy controls;

† differences between patients and age-matched healthy controls.

**Table 2.** Swine characteristics

	Baseline (*n* = 7)	Post-MI (*n* = 6)
Heart rate, beats/min	76 ± 15	95 ± 9.4*
Systolic blood pressure, mmHg	81 ± 7.2	89 ± 7.1
Diastolic blood pressure, mmHg	45 ± 8.3	48 ± 8.3
LV end-diastolic volume, mL	107 ± 9.8	110 ± 7.3
LV end-systolic volume, mL	52 ± 6.1	52 ± 4.7
LV stroke volume, mL	55 ± 10	58 ± 6.8
Longitudinal contribution to SV, mL	30 ± 6.3	33 ± 5.2
Longitudinal contribution to SV, %	56 ± 8.0	57 ± 8.1
Radial contribution to SV, mL	25 ± 6.6	25 ± 5.9
Radial contribution to SV, %	44 ± 8.0	43 ± 8.1
Ejection fraction, %	51 ± 6.3	52 ± 4.0
Cardiac output, L/min	4.2 ± 1.0	5.5 ± 0.8*
Rate pressure product, mmHg/min	6,419 ± 1,209	8,800 ± 933*
Max AV-plane displacement, mm	9.3 ± 1.7	9.9 ± 0.9
Max average epicardial displacement, mm	1.6 ± 0.4	1.9 ± 0.5
End-diastolic longitudinal epicardial surface area, cm^2^	34 ± 2.8	35 ± 2.5
End-diastolic radial endocardial surface area, cm^2^	104 ± 6.1	105 ± 5.5
Peak LV pressure (invasive measure), mmHg	79 ± 4.8	80 ± 5.9

Continuous variables are reported as means ± SD. MI, myocardial infarction; LV, left ventricular; AV, atrioventricular.

* Significant differences (*P* < 0.05) from baseline cohort.

### 

#### Experimental validation.

**Fig. 5.**
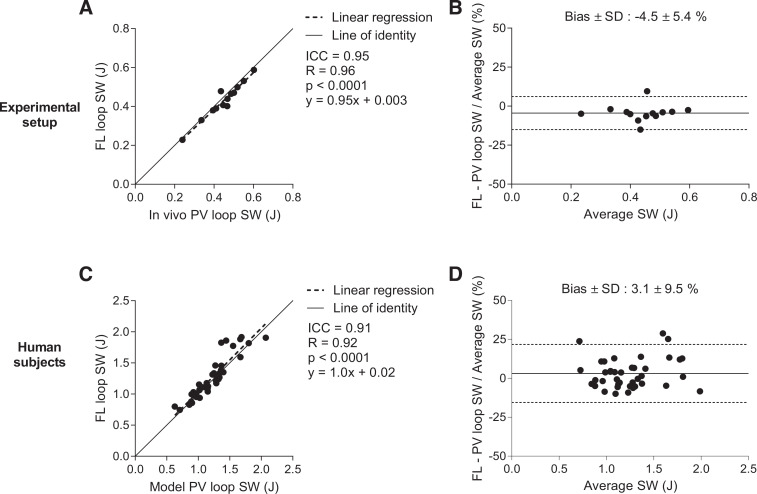
Validation of stroke work (SW) derived from force-length (FL) loops compared with SW derived from pressure-volume (PV) loops. Invasively measured PV loops were used in the experimental setup (*top row*), and model-based noninvasive PV loops in the human participants (*bottom row*). The FL loop-derived SW was calculated as the sum of the area within the longitudinal and radial FL loops. *A*: scatter plot for the experimental setup. *B*: Bland–Altman plot for the experimental setup. Solid line represents the bias and dashed lined the limits of agreement. *C*: scatter plot for human participants. *D*: Bland–Altman plot for human participants. Swine had smaller ventricles and lower pressure compared with humans, which explains the differences in SW values between the cohorts. ICC, intraclass correlation coefficient.

There was excellent agreement and low bias in SW measured from pressure-volume loops and SW calculated as the sum of longitudinal and radial force-length loop areas in swine, as shown in Fig. 5, *A* and *B*. This suggests that the proposed method for calculating the longitudinal and radial contributions to SW using force-length loops holds.

Table 3 summarizes the experimental peak force and total, longitudinal, and radial SW, as well as WEV, for swine at baseline and post-myocardial infarction. Longitudinal and radial pumping were found to contribute approximately equally to SW in the entire experimental cohort (51 ± 6.4 and 49 ± 6.4%, respectively). There was no difference in longitudinal (*P* = 0.84) or radial (*P* = 0.84) contributions to SW between swine at baseline and post-myocardial infarction.

**Table 3.** Experimental results

	Baseline (*n* = 7)	Post-MI (*n* = 6)
Peak longitudinal force, N	30 ± 3.6	35 ± 3.7
Peak radial force, N	88 ± 9.3	100 ± 9.4
Total SW from PV loop, J	0.41 ± 0.1	0.49 ± 0.1
Longitudinal SW, J	0.19 ± 0.05	0.24 ± 0.03
Longitudinal SW, %	49 ± 6.9	52 ± 6.0
Radial SW, J	0.20 ± 0.06	0.23 ± 0.06
Radial SW, %	51 ± 6.9	48 ± 6.0
Longitudinal WEV, mJ/mL	6.6 ± 0.8	7.4 ± 0.4*
Radial WEV, mJ/mL	8.2 ± 0.9	9.4 ± 1.3

Continuous variables are reported as means ± SD. MI, myocardial infarction; LV, left ventricular; SW, stroke work; PV, pressure-volume; WEV, work per ejected volume.

* Significant differences (*P* < 0.05) from baseline cohort.

Longitudinal WEV was lower than radial WEV (6.8 ± 0.8 vs. 8.7 ± 1.2 mJ/mL, *P* = 0.0002). Furthermore, longitudinal WEV was lower at baseline compared with 1 wk postinfarction (*P* = 0.01), but differences in radial WEV did not reach statistical significance (*P* = 0.07). With equal contributions to SW but not stroke volume, this indicates that the longitudinal mode of pumping is more energy efficient than the radial pumping in delivering stroke volume. In the swine cohort, this level of efficiency in longitudinal pumping was altered 1 wk post-myocardial infarction, but it remained more efficient than radial pumping.

#### Cohort comparisons.

Table 4 summarizes peak force and longitudinal and radial contributions to SW and WEV in all human participants, disclosing no differences between young healthy controls and older controls. There were, however, differences between patients and age-matched controls in peak longitudinal and radial force as well as in the longitudinal and radial contributions to SW.

**Table 4.** Results in human participants

	Young Healthy Controls (*n* = 12)	Age-Matched Healthy Controls (*n* = 14)	Patients (*n* = 14)
Peak longitudinal force, N	56 ± 10	61 ± 9.0	91 ± 20*†
Peak radial force, N	188 ± 38	180 ± 22	269 ± 56*†
Total SW from PV loop, J	1.3 ± 0.3	1.2 ± 0.2	1.2 ± 0.3
Longitudinal SW, J	0.65 ± 0.2	0.61 ± 0.13	0.59 ± 0.2
Longitudinal SW, %	49 ± 4.0	53 ± 4.9	44 ± 5.6†
Radial SW, J	0.70 ± 0.2	0.53 ± 0.1	0.72 ± 0.2†
Radial SW, %	51 ± 4.0	47 ± 4.9	56 ± 5.6†
Longitudinal WEV, mJ/mL	9.8 ± 1.4	11 ± 0.8	12 ± 3.0*
Radial WEV, mJ/mL	15 ± 4.3	17 ± 4.2	17 ± 4.2

Continuous variables are reported as means ± SD. SW, stroke work; PV, pressure-volume; WEV, work per ejected volume.

* Significant differences (*P* < 0.05) from young healthy controls;

† differences between patients and age-matched healthy controls.

In the healthy controls and patients, the calculation of longitudinal and radial contributions to SW using force-length loops yielded excellent agreement and low bias compared with SW derived from noninvasive pressure-volume loops, as shown in Fig. 5, *C* and *D*.

**Fig. 6.**
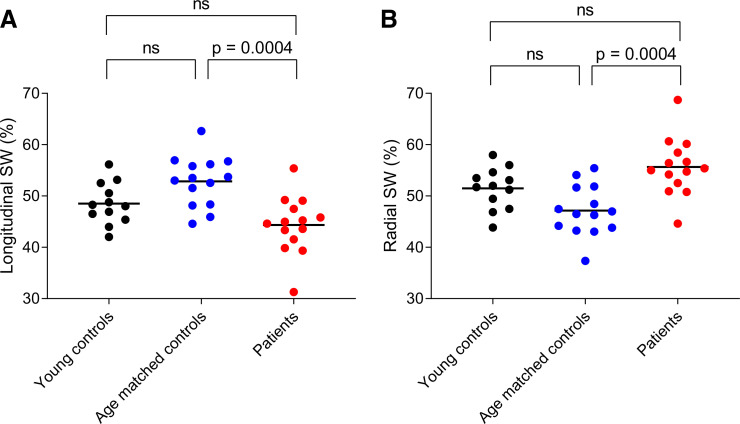
Comparison of longitudinal and radial stroke work (SW) in the human cohorts. Young healthy controls are shown in black, age-matched healthy controls in blue, and patients in red. *A*: longitudinal contribution to SW for young controls, age-matched controls, and patients. *B*: radial contribution to SW for young controls, age-matched controls, and patients.

Longitudinal and radial pumping contributed approximately equally to SW in young healthy controls (49 ± 4.0 and 51 ± 4.0%, *P* = 0.23, respectively) and age-matched healthy controls (53 ± 4.9 and 47 ± 4.9%, *P* = 0.02, respectively), as shown in Fig. 6. In the patients, there was a slight shift in work distribution from longitudinal to radial pumping (44 ± 5.6 and 56 ± 5.6%, *P* = 0.002, respectively). This was due to dilated ventricles and thus larger surface areas, as well as decreased AV-plane and epicardial displacements in the diseased hearts.

**Fig. 7.**
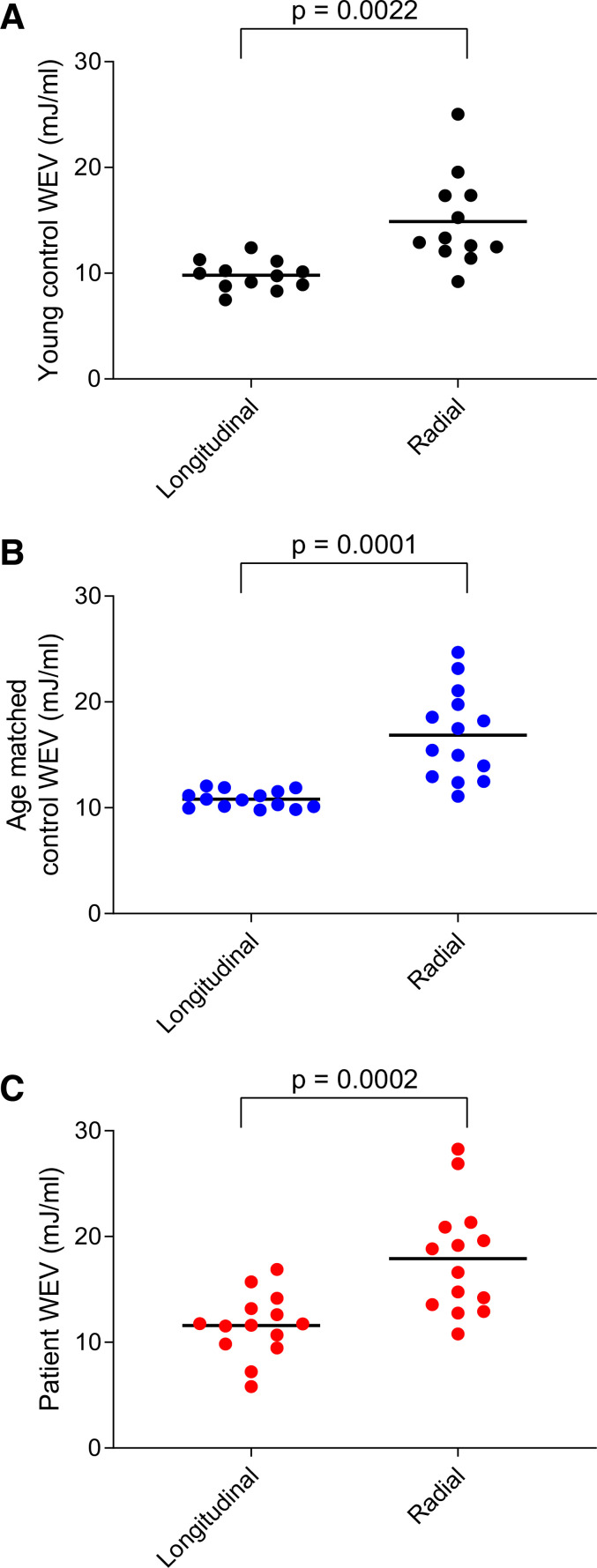
Comparison of longitudinal and radial work per ejected volume (WEV) in the human cohorts. Young healthy controls are shown in black, age-matched healthy controls in blue, and patients in red. *A*: longitudinal and radial WEV in young healthy controls. *B*: longitudinal and radial WEV in age-matched healthy controls. *C*: longitudinal and radial WEV in patients.

Longitudinal WEV was lower than radial WEV in young healthy controls, age-matched healthy controls, and patients (Fig. 7). Longitudinal WEV was also lower than radial WEV when averaged over all participants (11 ± 2.1 vs. 17 ± 4.7 mJ/mL, *P* < 0.0001), indicating that longitudinal pumping was more energy efficient than radial pumping in delivering stroke volume in the human cohort as well as in the swine cohort. Longitudinal WEV was lower in young healthy controls compared with patients (*P* = 0.05), but there was no difference between young and age-matched controls (*P* = 0.26) or between age-matched controls and patients (*P* > 0.99). There was also no difference in radial WEV within the human cohorts.

#### Sensitivity analysis.

The noninvasively PV loop-derived SW increased exponentially with increasing V_0_, with an average difference of 2.5 ± 0.8 J between the SW calculated for V_0_ = 0 and for V_0_ = 0.9·ESV. The longitudinal and radial contributions to SW in percent remained essentially constant, with an average difference of 0.006 ± 0.02% between the longitudinal contribution to SW in percent for V_0_ = 0 and V_0_ = 0.9·ESV and −0.006 ± 0.02% for the radial contribution. This means that the noninvasively derived relative longitudinal and radial contributions to stroke work in percent holds without a subjects-specifically determined V_0_, whereas the quantitative contributions in Joules might not be accurate in patients with V_0_ significantly larger than zero.

## DISCUSSION

This study presents a validated methodology for quantification of longitudinal and radial contributions to SW by using global ventricular force-length loops. The major finding was that the longitudinal and radial contributions to SW is approximately equal in healthy controls, but not in patients with dilated ischemic cardiomyopathy, and that the longitudinal mode of pumping is more energy efficient than radial pumping in delivering stroke volume. These insights expand the physiological understanding of LV SW and energy consumption and could potentially be of value in novel clinical applications.

Longitudinal and radial pumping has previously been shown to account for ~60% and ~40%, respectively, of the LV stroke volume, which was confirmed in this study (5, 6). The contribution to SW, however, had not been investigated previously as it is measured from pressure-volume loops, which do not contain intrinsic information on longitudinal and radial function. In this study, we found that longitudinal and radial contributions to SW can be achieved by instead quantifying work as the area within one force-length loop for each mode of pumping. By defining length as the tissue displacement in the respective directions and studying the force applied on the surface area that the blood pressure interacts with, longitudinal and radial force-length loops were derived. The sum of the work derived from these force-length loops showed excellent agreement with SW quantified conventionally from pressure-volume loops.

The ability to derive force-length loops noninvasively is important for potential clinical applicability, as invasive pressure catheterizations impose risk on patients. In this study, noninvasive quantification of force-length loops was shown to be feasible using a recently published method for noninvasive quantification of LV pressure from CMR and brachial cuff pressure (36), as the agreement between force-length loop- and pressure-volume loop-derived SW was strong using this novel pressure quantification. For the noninvasive pressure estimation, end-diastolic pressure was set to 5 mmHg in all human participants. This value is not subject-specifically representative and will influence the estimated pressure curve, but a sensitivity analysis has previously shown that the impact on quantitative SW is low when varying the end-diastolic pressure between 0 and 15 mmHg (36). Importantly, the value of end-diastolic pressure will not impact the calculated proportions of longitudinal and radial contributions to SW.

Longitudinal and radial functions were shown to contribute ~50/50% to SW in human healthy controls and 44/56% in heart failure patients. With a dominating longitudinal contribution to stroke volume of ~60 vs. ~40% (5, 6), this finding may at first be surprising. However, when the WEV was considered, longitudinal pumping was found to be more energy efficient than radial pumping. This finding is consistent with previous work by Ghosh et al. (11), who, by quantifying longitudinal and radial LV impedances, showed that the physiological preference for LV filling in early diastole is longitudinal volume accommodation. The level of energy efficiency in delivering stroke volume was slightly lower in swine one week post myocardial infarction compared with baseline experiments. It is, however, important to point out that the swine were not their own controls, and it is therefore not possible to draw any conclusions on alterations in pumping mechanisms on an individual level. Human patients with dilated ischemic cardiomyopathy had a lower level of energy efficiency in delivering stroke volume compared with young healthy controls but not compared with age-matched healthy controls. Moreover, the patients in this study had stable chronic heart failure for which they were all medicated. In heart failure without a combination of, for example, β-blockers, ACE inhibitors, and diuretics, patients would be expected to have volume-loaded left ventricles with more stretched sarcomeres and thus positioned farther to the right in the Frank–Starling curve. How this would impact force-length loops and the longitudinal and radial contributions to stroke work is unknown.

Many clinical markers of cardiac motion are longitudinal, for example AV-plane displacement, MAPSE, peak systolic and early diastolic mitral valve velocities (s′, e′), and global longitudinal strain (16, 17, 25, 30, 31). Few clinical parameters are radial, however, in part due to poor reproducibility of, for example, global radial strain (2). The findings in this study give further merit to parameters reflecting the longitudinal mode of pumping, as they explain ~50% of the SW performed in the studied cohorts. This might be a reason for the strong prognostic value of these longitudinal clinical markers (3, 30, 31, 33, 47), which are thus important in both healthy controls and heart failure patients. The physiological insights on the LV work and energy consumption could furthermore be potentially of value in the development of novel cardiac assistance devices and in tissue engineering aiming at restoring the myocardial pump function in an energy-efficient manner.

The full picture translating from the local orientation of cardiomyocytes to global anatomic geometry and its coupling with blood flow is complex, however, involving several different forces. The forces calculated in this study should be interpreted as counterforces balancing the longitudinal and radial force components generated by the contracting myocardium on a global level, according to Newton’s third law. The forces should not be confused with the closely related metric wall stress (in N/m^2^), i.e., the average myocardial tension, which is determined from the pressure, wall thickness, and LV radius (24). Additional balancing forces between blood and the myocardial wall are present over the cardiac cycle – in the apical direction, for example. However, remembering that work is performed only if there is a change in length or an active displacement, the essentially static apex does not perform any work. Additional forms of cardiac energy consumption not contributing to external ventricular work, such as mechanical potential energy and kinetic energy of the blood, were not studied. A comprehensive description of intracardiac forces remains to be investigated, and understanding the contribution to longitudinal and radial work might be a step in that direction.

It is important to distinguish between force (pressure multiplied by area) and work (force × displacement). Since work requires a displacement, this study considered only the surfaces of moving myocardium in the respective longitudinal and radial directions in the work calculations. Of note, all layers in three-dimensional myocyte architecture contribute to both longitudinal and radial shortening (12, 35). This means that the radial displacement does not originate exclusively in the contraction of myocytes in the epicardial layer. Epicardial displacement can, however, from a global perspective be interpreted as the net radial displacement due to myocardial contraction, since an isolated longitudinal shortening in all layers would not contribute to an inward epicardial movement (Fig. 3). The inward movement of the endocardial layer is, on the other hand, a result of both longitudinal and radial shortening. Thus, endocardial displacement was not a suitable reference point for measuring displacements in this study, as the portions of displacement due to each mode of pumping could not be isolated and quantified from the acquired images in this study. Apart from longitudinal and radial shortening, there is also a twisting myocardial motion that contributes significantly to the ejection fraction (34). Twisting and untwisting are the results of the interaction between individual angulated myocytes and their three-dimensional architecture, and the motion is similar to the one originated when a wet towel is wrung out (40). Although the proposed model in this study did not separately calculate the twisting contribution to SW, its contribution is encompassed in the presented concepts of longitudinal and radial work. Going back to the wet towel, the wringing will on a local level rearrange the fibers of the towel into a twisted architecture. From a more global perspective, the wringing results in an overall shortening of the towel, which can be observed as a decrease in length and radius of its outer boundaries. Hence, twisting accounts for part of the AV-plane and epicardial displacement and is therefore included as a part of the longitudinal and radial contributions to the SW proposed in this study. This explains why the longitudinal and radial SW adds up to ~100% of the PV loop-derived SW without explicitly taking twisting into account.

### 

#### Limitations.

The proposed method is a simplified conceptual model of LV function, and it is important to note that all cardiomyocytes and sheetlets contribute simultaneously to ventricular shortening in both the longitudinal and the radial directions. Longitudinal and radial pumping should therefore not be interpreted as two independent functionalities that the heart can turn on and off separately but rather as two coupled modes of pumping describing global ventricular function. In-depth simulations of longitudinal and radial work at the cardiomyocyte level can be attained using previously-proposed multiscale numerical models that incorporate the orientation and mechanoelastic components of the cardiomyocytes (1, 9, 44, 45): by calculating stress-strain relationships, for example. These models are complex, however, and computationally demanding, and the myocardial microstructure cannot be subject-specifically determined (26). The global simplified framework proposed in this study is therefore a more applicable method to provide insights into subject-specific quantitative longitudinal and radial LV energy consumption. It also allows the study of longitudinal and radial force-length loops in larger cohorts and from this derive potential prognostic information.

Validation of the framework was performed by comparing force-length loop- and pressure-volume loop-derived SW. Pressure-volume loops were derived using LV pressure and endocardial delineations, and the same measurements were also used in the calculations of longitudinal and radial force-length loops. There is thus an overlap in the measurements used in the proposed method and the reference method, introducing bias into the validation. This bias is, however, partially circumvented, since the proposed force-length loops cannot be generated without epicardial delineations and AV-plane displacement, measurements that are not accounted for in the pressure-volume loops.

The method to obtain noninvasive pressure-volume loop has been validated in swine with the equilibrium volume V_0_ approximated to zero, and further validation of the method and its model parameters in humans is warranted. However, the method is expected to translate to humans, as it is based on the time-varying elastance, which has been shown to have the same fundamental shape regardless of several pathological states and in a variety of mammalian species (10, 38, 39). Ky et al. (20) have furthermore shown that V_0_ can be significantly larger than zero in chronic heart failure patients. The relative longitudinal and radial contributions to SW in percentages did, however, remain essentially constant when a sensitivity analysis was performed with regard to V_0_, meaning that the approximation V_0_ = 0 holds for these calculations. Caution should be taken, however, when interpreting the quantitative hemodynamic parameters derived from the noninvasive pressure-volume loop model in chronic heart failure patients, which may experience a greater impact if there is a large discrepancy between the actual and zero-approximated V_0_.

#### Conclusions.

In conclusion, this study found that the longitudinal and radial contributions to SW are ~50/50% in healthy controls. This distribution is slightly altered to ~45% longitudinal and ~55% radial contribution to SW in heart failure patients due to dilated ischemic cardiomyopathy. Furthermore, the longitudinal mode of pumping is more energy efficient in delivering stroke volume. Further studies are warranted to evaluate the clinical role of the proposed method.

## GRANTS

This study was funded by the Swedish Research Council (2017-04389), the Swedish Heart Lung Foundation (20170554), the Wallenberg Centre for Molecular Medicine at Lund University, the ALF Medical Faculty Grants at Lund University (17401, 446881, 442941, and 2014354), and Vinnova (2017-01451).

## DISCLOSURES

E. Heiberg is the founder of Medviso AB, Lund, Sweden, the company providing a commercial version of Segment. J. Berg and K. Solem are employees of Syntach AB, Lund, Sweden. None of the other authors has any conflicts of interest, financial or otherwise, to disclose.

## AUTHOR CONTRIBUTIONS

F.S., K.S., H.A., M.C., and E.H. conceived and designed research; F.S., J.B., K.S., and R.J. performed experiments; F.S. analyzed data; F.S., J.B., K.S., R.J., H.A., M.C., and E.H. interpreted results of experiments; F.S. prepared figures; F.S. drafted manuscript; F.S., J.B., K.S., R.J., H.A., M.C., and E.H. edited and revised manuscript; F.S., J.B., K.S., R.J., H.A., M.C., and E.H. approved final version of manuscript.
